# Protective effect of KIR3DS1 in asymptomatic HIV-1 seroconverters towards AIDS

**DOI:** 10.1186/1471-2334-12-S1-P69

**Published:** 2012-05-04

**Authors:** M Stalinraja, N Kalyanaraman, P Leishman, M Suresh, M Jayalakshmi

**Affiliations:** 1 Dept of Immunology, School of Biological Sciences, Madurai Kamaraj University, Madurai - 21, India; 2 ART Centre, Govt Theni Medical College and Hospital, Theni, India

## Background

The extreme variability at the Killer cell Immunoglobulin like Receptor (KIR) expressed on the surface of natural killer cells interacts with Human Leukocyte Antigen (HLA) Class I appear to play a crucial role in the outcome against viral infection. The combination of HLA/KIR gene products either individually or collectively have been implicated in the control of HIV-1 in various populations. Objective of this immunogenetic case-control study is to determine the association between KIR3DS1 and HIV-1 asymptomatism.

## Methods

31 treatment naive HIV-1 asymptomatic (>3yrs without disease progression) and 31 HIV-1 seronegative ethnic matched healthy controls were recruited for this case – control study. Genomic DNA was extracted from peripheral blood by salting out procedure and KIR genotyping was performed for 16 KIR genes (Activating, Inhibitory and Pseudogenes) by Duplex PCR sequence-specific primer method.

## Results

Overall, we observed 66.12 % of individuals with B KIR haplotype among case and controls group. Among the 16 KIR genes were typed KIR3DS1 (Activating KIR gene) shows significant variation among HIV asymptomatic individuals was 74.2 % whereas 54.8 % among controls.

## Conclusion

Since, KIR3DS1 is well known for its effect on direct viral containment, presence of higher KIR3DS1 is one of the possible reasons for asymptomatism among this study cohort.

